# Outline of a Scheme for Detection and Treatment

**Published:** 1913-08-30

**Authors:** G. J. Masson Martin

**Affiliations:** Junior School Medical Officer, Portsmouth.


### THE SCHOOL MEDICAL OFFICER AND TUBERCULOSIS.

### Outline of a Scheme for Detection and Treatment.

By G. J. MASSON MARTIN, Junior School Medical Officer, Portsmouth.

The function of the school medical officer is twofold—first, to recognise and correct any disease which prevents the attendance and consequently the immediate education of the child at school; and, secondly, to diagnose and remedy any defect which may, in after-life, prevent him becoming a useful member of society. The first of these objects is usually fairly easily obtained, skin diseases and other defects which cause temporary absence from school being, as a rule, cleared up by energetic treatment; but the diagnosis and cure of diseases which may cause ill-health in after-life often present much greater difficulty.

Tuberculosis, a few years ago, was considered to be a very rare disease in childhood, but the recent researches of Continental observers show the contrary to be the case, it now being known to be the most common disease to which children of the proletariat are liable. By the specific test Hamburger and Monti have shown that tuberculosis is present in 20 per cent. of school children in Vienna in their third year, in 70 per cent. between the ages of seven and eight, and in 90 per cent. in children of fourteen years of age; and these figures have been substantiated and confirmed by many other observers for other towns of the Continent.* These statistics go to prove that the majority of, if not all, cases of tuberculosis showing themselves in adults must be due to a primary infection during childhood; and of all diseases tuberculosis is the most noted in rendering a great number of the adults of the poorer classes useless members of the community, besides making them sources of infection to others.

### Its Incidence in School Children.

Incipient tuberculosis, then, is one of the diseases which it is the duty of the school medical officer to detect and remedy, it being a disease which occurs primarily in childhood, and one which is responsible for most of the chronic incapacitating illness and early death among the lower classes of the community.

Up to the present, however, no very great attempt has been made to detect this disease amongst school children in this country. Only cases which manifest themselves as definitely tuberculous, such as hip disease, "open" phthisis, etc., are notified and treated; but most school doctors must be struck by the number of cases they come across during their routine examination in which they find certain indefinite signs which suggest tuberculosis to be present. Although the examiner may have little doubt in his own mind that such children have a slight tubercular infection, the signs are not decisive enough to warrant him to notify the parents, and perhaps prevent the attendance of the child at school, by stamping him as definitely tubercular. The majority of such cases no doubt cure themselves, but a small proportion of them, in whom the infection is sufficiently virulent, or the resistance of the patient sufficiently weak, form the number in whom the disease culminates after or during puberty.

As tuberculosis is a disease which we now know to be capable of cure, or at least arrest, during its early stages, if it were practicable for the school medical officer to detect and place such cases in circumstances which would conduce to their cure during childhood or early youth, it would seem that in this manner the occurrence of the disease in adult life would be most surely and effectively prevented.

### The Tuberculin Test.

The only certain means we have of demonstrating the presence of a tuberculous focus in the body is by the specific test by tuberculin. There are, as is well known, four methods of applying this test—Moro's, or the percutaneous reaction; Von Pirquet's, or the cutaneous reaction; Calmette's, or the conjunctival test; and the subcutaneous reaction as used originally by Koch. Of these methods the most suitable for the diagnosis of the disease in school children is undoubtedly the cutaneous reaction of Von Pirquet. The eye and subcutaneous reactions, although reliable as diagnostics, are not altogether free from danger, and the percutaneous reaction of Moro is not sufficiently trustworthy in its results. If, however, 70 to 90 per cent. of children respond to the tuberculin test, a positive result will not be found to be of much value as an indication for a call for immediate treatment; but a recent modification of Von Pirquet's test elaborated by two Danish observers—Ellerman and Erlandsen—renders it of very great value in the above respect. The method has been described by Dr. E. C. Morland (*Lancet*, 1912, 2, p. 688) and has been named the "Quantitative Cutaneous Tuberculin Test" (Quanti-Pirquet). By means of this test it is claimed that the question of the necessity or otherwise of treating a tuberculin-sensitive individual is solved. By an ingenious system of comparative measurement of the size of the papules obtained after scarification of the skin through drops of tuberculin of different strengths, a figure is arrived at which indicates the tuberculin-sensitiveness of the subject. This sensitive value having been ascertained, its clinical significance is interpreted by the experience of the authors and others, in that those cases which give results above a certain figure show need of treatment, and those below this figure generally do not—that is, they show sufficient resistance to enable the body to overcome the disease of itself under ordinary healthy conditions. The value of this test, if applied to school children, is obvious, but, of course, in deciding the necessity or otherwise for treatment the circumstances and physical condition of the child and many other factors must be taken into consideration, besides the tuberculin sensitiveness, before one can arrive at a conclusion.

While admitting the value of such a test for school children, some may argue that although very desirable and capable of good results in theory, it would be impossible to put it into practice in this country owing to the resistance of the parents to what they might consider an "experiment" or "operation" on their children, and their consequent, not altogether unnatural, objection to have their children tampered with. As soon as it was found, however, that the test was harmless in its results, and not carried out in a spirit of meddlesome bureaucracy, but for the future welfare of the child, it would be found that the majority of the parents would be only too glad to facilitate the putting of it into practice. The dire results of such a disease which the poor have continually before their eyes would add materially to their anxiety to aid the medical officers in saving their children from a similar fate.

### Treatment During School Career.

It is assumed that the parents of such children are unable to pay for their medical attendance—that they are of the same class, in short, which would be entitled to tuberculosis benefit under the Insurance Act.

The best time during school life for the application of this test would be at the two later periods of routine examination—that is, children between seven and eight years and "leavers," or those between twelve and fourteen years old.

With regard to the question of cure, the treatment of "leavers" would pass out of the hands of the school doctor, and the positive results of the test would be handed over to the tuberculosis authorities, and such authorities left to deal with these cases as they thought fit. Those children which showed necessity for treatment between seven and eight years would, however, need to be looked after by the school medical officers to the end of their school career. This treatment could be carried out under the usual heads, dietetic, hygienic, medicinal, and specific—modified, of course, by the exigencies and limitations of school and home life. The dietetic would include, if necessary, the provision of special free meals, the hygienic the sending of the child to an open-air school, the medicinal the provision of tonics, etc., and the specific the treatment by tuberculin or other method if the case were considered to be a fit one for such treatment.

### A Summarised Procedure.

In short, the diagnosis and treatment of incipient tuberculosis amongst board-school children by school medical officers might be summed up in the following outline of procedure:—

### For Children Between 7 and 8 Years.

1. If during routine examination the slightest suspicion is aroused in the mind of the examiner of the presence of tuberculosis in a child, by certain indefinite signs in the chest or elsewhere, coupled with morning anorexia, lassitude, malnutrition, night sweats, nervous symptoms, etc., a notice should be sent the parents informing them that a further examination and test is necessary to determine the presence or absence of disease in their child. Evidence should also be sought, before examination, from the parents, of any family history of tuberculosis or the presence of a case of tuberculosis in the same house as the child, and the test applied to all such "contact" cases.
2. Application of Quanti-Pirquet test.†
3. Interpretation of result.
4. Notice to parents indicating either (1) the freedom of the child from disease; (2) the presence of a "tendency towards tuberculosis," but the non-necessity of its treatment beyond the exercise of ordinary open-air hygienic measures at home (such measures to be shortly described); or (3) the presence of tuberculosis and the necessity for its treatment.
5. The carrying out of treatment (1) dietetic, (2) hygienic, (3) medicinal, and (4) specific, by (1) if necessary special free meals, (2) open-air school and leaflets to parents describing home sanatorium methods, (3) drugs, and (4) tuberculin or other method if thought suitable. The procedure in cases of children between twelve and fourteen will be the same, except that after the test has been carried out the result, if positive, would be sent to the tuberculosis authorities and the further treatment of the case left in their hands; or the child could be sent to them for the test if this method were found more mutually suitable.

The above does not pretend to be anything more than a bare outline of a scheme to be carried out by school medical officers. It is only a suggestion of a plan to those who—when we know now that tuberculosis almost invariably starts in childhood—have in their hands the greatest opportunity of stamping it out, greater than any other public health authority, seeing that school doctors have only to destroy the seedling and not to cut down the full-grown tree, as is the duty of the tuberculosis officer and others. It seems to the writer that some such scheme, if rigorously carried out all over the country, would be one of the most potent means of ridding the community of a malady which is such a constant drain on the present social economy, and a little money spent at present in this way would mean the saving of many thousands in coming years. It is easier and less costly to quench a spark than to endeavour to smother a roaring conflagration.

* In Paris by Mantoux, in Düsseldorf by Nothmann, and in Prague by Ganghopner.

† Messrs. Allen and Hanbury supply a small outfit with directions for this test, price 27s. 6d.

